# Sex-specific differences in the anatomical localization and severity of coronary artery disease: differential impact of conventional cardiovascular risk factors

**DOI:** 10.1016/j.clinsp.2026.101149

**Published:** 2026-09-15

**Authors:** Nurgül Keser, Yetkin Korkmaz, Selami Doğan, Mehmet Şeker, Samet Yavuz, Mert Babaoğlu, Almina Erdem, İrem Yılmaz, Mustafa Oğuz, Akın Torun, Lutfullah Orhan, Mehmet Uzun

**Affiliations:** a Hamidiye Faculty of Medicine, University of Health Sciences, Istanbul, Turkey; b Sultan Abdülhamid Han Training and Research Hospital, Ministry of Health of the Republic of Turkey, Istanbul, Turkey

**Keywords:** Risk factors, Atherosclerosis, Coronary artery obstruction, Sex

## Abstract

**Background:**

Sex differences in coronary artery disease (CAD) are well recognized. Whether conventional cardiovascular risk factors (CVRFs) exert differential effects on anatomical localization and stenosis severity on coronary arteries (CA) remains uncertain. We aimed to evaluate sex-specific associations between major CVRFs and CA involvement.

**Methods:**

We analyzed 978 patients undergoing coronary angiography (CAG) between 2017–2023. Sex, age, hypertension (HT), diabetes mellitus (DM), history of CAD (HCAD), smoking, Syntax scores (SS), lipid parameters and creatinine levels were assessed. Segment-based coronary involvement was evaluated using correlation and multivariable regression analyses.

**Results:**

Women were older (p < 0.001), had higher rates of HT (p = 0.001), DM (p = 0.026) and higher total cholesterol (p = 0.001), LDL-C (p = 0.001) and HDL-C levels (p = 0.001). In contrast HCAD (p = 0.001), smoking (p = 0.009) and SS (p < 0,01) were higher in men. HT was associated with circumflex (Cx) (p = 0.029) and right coronary artery (RCA) (p = 0.002) involvement in women; with proximal left anterior descending artery (LAD) (p = 0.024) involvement in men. DM showed significant associations with LAD (p = 0.001), 1st diagonal (Dg) (p = 0.003), Cx: (p = 0.001) and RCA (p = 0.001) territories in women. HCAD was associated with proximal LAD (p = 0.013), CX (p = 0.006) and RCA (p = 0.016) in men; proximal LAD (p = 0.047), CX (p = 0.014) and RCA (p = 0.009) in women. Lipid parameters had no association with CA involvement in either sex after adjustment. Creatinine levels were associated with RCA and CX involvement in women and with LMCA and proximal LAD territories in men.

**Conclusion:**

CVRFs exert sex-specific effects on CA, highlighting the importance of sex aware risk stratification in CAD.

## Highlights

• There are sex-based differences between Cardiovascular Risk Factors (CVRF).
• There are sex differences in the impact of CVRF on coronary arteries.
• Correlation between CVRF and coronary artery obstruction needs further evaluation.
• Sex-specific strategies in coronary artery disease management is needed.

## Introduction

Coronary Artery Disease (CAD) remains the leading cause of mortality and morbidity worldwide in both sexes^1^ Numerous studies have demonstrated important sex-based differences in Cardiovascular Risk Factors (CVRFs),^2^^,^^3^ the development and progression of atherosclerosis, plaque characteristics, clinical presentation, diagnosis, therapeutic strategies, and preventive approaches.^4^, ^5^, ^6^ Nonobstructive CAD and microvascular coronary dysfunction are also well recognized to be more prevalent in women.^5^ In addition, there is a clear difference in the age of disease onset with men more frequently affected at younger ages, whereas women tend to experience a greater disease burden after menopause.^7^, ^8^, ^9^, ^10^

Despite these well-established sex differences, currently available risk scoring systems used to predict and assess CAD are largely identical for men and women. This lack of advanced sex-specific risk stratification, combined with limited awareness of sex differences in CAD among physicians and among the general population, may contribute to the underdiagnosis and undertreatment of CAD in women.

## Objectives

In this study, we compared major conventional CVRFs between men and women and examined their associations with the anatomical localization and severity of obstruction in epicardial coronary arteries in order to investigate potential sex specific effects of each CVRF on CAD burden and to inform the necessity of development of novel risk scoring systems that incorporate the sex specific impact of individual CVRFs in the era of precision medicine.

## Methods

### Study population

This study was designed as a single-center, retrospective, descriptive study. A total of 978 patients aged ≥ 20-years, including 488 women, who underwent diagnostic Coronary Angiography (CAG) due to clinical symptoms and abnormal non-invasive test results at our institution between 2017 and 2023 were included.

Patients with incomplete or unavailable demographic data, medical records, echocardiographic findings, or laboratory parameters, as well as those with a history of CABG/valvular surgery or prior PCI/stent implantation, were excluded. None of the female patients consumed hormone replacement therapy.

Incomplete data were addressed by complete case analysis, excluding observations with missing values. This approach was chosen because the proportion of missing data was low (< 5%) and missingness was assessed to be completely at random. As this was a retrospective descriptive study, STROBE flow was followed as below.

### Data collection

Eligibility for inclusion and data collection were determined by a cardiologist, based on physical examination findings, transthoracic echocardiography, ECG, key laboratory parameters, and clinical data. Laboratory data included fasting blood glucose (glucose), creatinine, Total Cholesterol (TC), LDL Cholesterol (LDL-C), HDL Cholesterol (HDL-C) and Triglyceride (TG) levels and clinical data included age, sex, Body Mass Index (BMI), smoking status, prevalence of Hypertension (HT), Diabetes Mellitus (DM), History of CAD (H.CAD) and Family history of premature CAD (F.CAD) all of which were obtained from the hospital medical records.

Biochemical analyses were performed on fasting blood samples collected during hospitalization using a Beckman Coulter LH 780 hematological analyzer and Beckman Coulter LH 780 equipment (Beckman Coulter, Brea, CA, USA).

Body weight (kg) and height (m) of each patient were recorded from the database and body mass index (kg/m^2^) was calculated as weight divided by height squared (m^2^).

HT was defined as a previous physician diagnosis of HT; current use of antihypertensive medication or systolic/ diastolic blood pressure ≥140/90 mmHg on repeated measurements.^11^

DM was defined as a previous physician diagnosis of DM; current treatment with insulin or oral antidiabetic agents, fasting plasma glucose ≥126 mg/dL and/or HbA1c value ≥ 6.5%.^12^

Dyslipidemia was defined as a previous physician diagnosis, serum TC ≥ 240 mg/dL, TG levels ≥ 200 mg/dL, LDLC ≥160 mg/dL, and current use of lipid-lowering medication.^13^

Self-reported smoking history was defined as either a current smoker who has been smoking >1-pack (20 cigarettes) per day for over 10-years or a former smokers who smoked 20-pack year in their lifetime and who were not an active smokers at the time of hospital admission, regardless of time since they quit.

F.CAD was defined as the presence of CAD in one or more first-degree relatives occurring before the age of 55-years in men and 65-years in women.^14^

H.CAD was defined as a previous diagnosis of CAD established by an experienced cardiologist using invasive and/or noninvasive diagnostic modalities.

### Analysis of coronary angiographies

All Coronary Angiograms (CAG) were visually analyzed to assess the severity of stenosis by two independent invasive cardiologists who were blinded to the patients’ detailed clinical and laboratory data. All vessels with a diameter > 1.5 mm were evaluated for stenosis severity.

Based on CAG images obtained using a GE Medical Systems workstation (SCS, France, AW volume share 2), Coronary Arteries (CA) were classified as Left Main Coronary Artery (LMCA), proximal Left Anterior Descending Artery (LAD), mid LAD, 1st Diagonal branch of LAD (Dg), Circumflex artery (CX), and Right Coronary Artery (RCA). Lesion severity in each coronary segment was categorized into three groups: 0: Mild (0%‒50%), 1: Moderate (50%‒70%), and 2: Severe (> 70%) vessel stenosis.

Finally, the SYNTAX score (version 2.28), which characterizes coronary anatomy according to the number of lesions involved, location and complexity of coronary lesions, was calculated for each patient as previously described in the literature.^15^ For the purpose of the present analysis, the mean SYNTAX Score (SS) obtained by the two independent observers was used.

### Statistical analysis

The NCSS (Number Cruncher Statistical System) 2020 (Kaysville, Utah, USA) program was used for statistical analysis. Descriptive statistical methods (mean, standard deviation, median, frequency, percentage, minimum, maximum) were used while evaluating the study data. The conformity of the quantitative data to the normal distribution was tested with the Shapiro-Wilk test and graphical examinations. Independent groups *t*-test was used for comparisons between two groups of normally distributed quantitative variables and the Mann-Whitney *U test* was used for comparisons between two groups of non-normally distributed quantitative variables. In order to evaluate the correlation between variables, Spearman’s Correlation analysis was used. Backward linear regression analysis was used to evaluate the effect of risk factors on the severity of obstruction.

Pearson Chi-Square test and Fisher-Freeman-Halton exact test were used to compare qualitative data. Statistical significance was accepted as p < 0.05.

## Results

### Comparison of demographic parameters and CVRFs

The study was conducted in our clinic with 978 patients between 2017 and 2023. 490 patients were male (50.1%), and 488 patients were female (49.9%). Women were older than men, with a mean age of 61 ± 10.3 years, whereas the mean age of men was 58 ± 10.8 (p < 0.001) years (Table 1). BMI was 28.74±4.7 in women and 28.52±4.6 in men, with no significant difference between the sexes (Table 1).

**Table 1:** Baseline demographic, clinical and laboratory characteristics of the study population.

**Variable**	**Men** **(n=490)**	**Women** **(n=488)**	**p-value**
Age (years)	58.0 ± 10.8	61.0 ± 10.3	a*0.001*
BMI (kg/m^2^)	28.5 ± 4.6	28.7 ± 4.7	a*0.71*
HT, n (%)	232 (47.3)	323 (66.2)	b*0.001*
DM, n (%)	165 (33.7)	198 (40.6)	b*0.026*
H-CAD, n (%)	182 (37.1)	109 (22.4)	b*0.001*
Smoking, n (%)	121 (24.7)	87 (17.8)	b*0.009*
F—CAD, n (%)	20 (4.1)	33 (6.8)	b*0.06*
Glucose (mg/dL)	125.3 ± 57.6	121.4 ± 57.5	c*0.47*
Creatinine (mg/dL)	1.52±7.03	1.08±3.96	c*0.001*
TG (mg/dL)	166.6 ± 134.3	147.4 ± 69.4	c*0.37*
HDL-C (mg/dL)	38.9 ± 10.6	49.1 ± 14.3	c*0.001*
Total-C (mg/dL)	183.0 ± 47.7	199.0 ± 44.4	a*0.001*
LDL-C (mg/dL)	112.2 ± 42.2	121.4 ± 37.0	a*0.001*

p < 0.01; p < 0.05.

BMI, Body Mass Index; HDL-C, High Density Lipoprotein; LDL-C, Low Density Lipoprotein; Total-C, Total cholesterol; TG, Triglyceride; H.CAD, History of Coronary Artery Disease; DM, Diabetes Mellitus; HT, Hypertension; F.CAD, Family history of Coronary Artery Disease.

a Student t-Test.

b Pearson Chi-Square Test.

c Mann Whitney-U-Test.

HT was present in 66.2% (n = 323) of women and 47.3% of men (n = 232) (p < 0.001) (p = 0.001; p < 0.01) (Table 1). DM was observed in 40.6% (n = 198) of women and 33.7% (n = 165) of men (p < 0.026) (p = 0.026; p < 0.05) (Table 1). Accordingly, HT and DM were found to be more prevalent in women.

H.CAD was present in 22.4% (n = 109) of women and 37.1% (n = 182) of men (p < 0.001). Smoking was reported by 17.8% (n = 208) of women and 24.7% (n = 121) of men (p < 0.009).

Thus, H.CAD and smoking history were more common in men, respectively as (p = 0.001; p < 0.01) and (p = 0.009; p < 0.01) (Table 1).

F.CAD was present in 6.8% (n = 33) of women and 4.1% (n = 20) of men (p > 0.05) with no significant difference between the groups (Table 1).

### Comparison of laboratory parameters by sex

TC, LDL-C and HDL-C levels were significantly higher in women, respectively, as (p = 0.001; p < 0.01, p = 0.001; p < 0.01, p = 0.001; p < 0.01) (Table 1). There were no significant differences in glucose or TG levels between men and women (p > 0.05) (Table 1). Although the difference in creatinine levels between sexes was statistically significant, being higher in men (p = 0.001; p < 0.01) the effect size and power were too small (0.077; 6.6%).

### Comparison of CAG findings between men and women

The incidence of critical stenosis, defined as ≥ 70% obstruction in any CA, was higher in men. Similarly, intermediate lesions defined as 50%‒69% vessel obstruction were more frequent in men. However, in Dg, no significant difference was observed in the degree of vessel obstruction between men and women (p = 0.357; p > 0.05) (Table 2).

**Table 2:** Distribution of stenosis severity across major epicardial coronary arteries by sex.

		**Men** **(n=490)**	**Women** **(n=488)**	**p-value**
**LMCA**	0	470 (96.0)	482 (98.8)	***0.017***^a^
	1	10 (2.0)	3 (0.6)	
	2	10 (2.0)	3 (0.6)	
**LAD proximal**	0	293 (59.8)	420 (86.1)	***0.001***^b^
	1	60 (12.2)	20 (4.1)	
	2	137 (28.0)	48 (9.8)	
**LAD Mid**	0	307 (62.7)	381 (78.1)	***0.001***^b^
	1	51 (10.4)	35 (7.2)	
	2	132 (26.9)	72 (14.8)	
**Dg**	0	402 (82.0)	416 (85.4)	***0.357***^b^
	1	30 (6.1)	24 (4.9)	
	2	58 (11.8)	47 (9.7)	
**CX**	0	280 (57.1)	374 (76.6)	***0.001***^b^
	1	72 (14.7)	34 (7.0)	
	2	138 (28.2)	80 (16.4)	
**RCA**	0	265 (54.1)	372 (76.2)	***0.001***^b^
	1	68 (13.9)	30 (6.1)	
	2	157 (32.0)	86 (17.6)	

LAD, Left Anterior Descending artery; LMCA, Left Main Coronary Artery; RCA, Right Coronary Artery; Dg, 1st Diagonal Artery; CX, Circumflex artery; 0, No stenosis, 1, Intermediate stenosis; 2, Severe stenosis (> 70%).

Fisher Freeman Halton Test.

a p < 0.05.

b p < 0.01.

The incidence of mild obstruction was significantly higher in women in the following vessels: LMCA (p = 0.017; p < 0.05), LAD prox (p = 0.001; p < 0.01), LAD mid (p = 0.001; p < 0.01), Cx (p = 0.001; p < 0.01) and RCA (p = 0.001; p < 0.01) (Table 2).

### Comparison of SS by sex

The mean SS was 4.03 ± 6.65 in women and 10.50±9.62 in men, with significantly higher values observed in men (p < 0.001) (Table 3). In patients with DM (p = 0.001; p < 0.01) and in those with a H.CAD (p = 0.001; p < 0.01), SS were significantly higher overall.

**Table 3:** Comparison of Syntax Score by sex and major cardiovascular risk factors.

		**Mean ± Sd**	**Median (IQR)**	**p**	**Post Hoc Effect size power**
**Sex**	Men	10.5 ± 9.6	8 (0‒44)	*0.001*^a^	0.783 power: 100%
	Women	4.0 ± 6.6	0 (0‒32)		
**HT**	(-)	7.0 ± 8.9	3 (0‒44)	*0.23*	0.052 power:5.1%
	(+)	7.5 ± 8.9	5 (0‒41)		
**DM**	(-)	6.2 ± 8.3	2 (0‒44)	*0.001*^a^	0.334 power: 99.9%
	(+)	9.2 ± 9.5	7 (0‒41)		
**H.CAD**	(-)	6.3 ± 8.8	2 (0‒44)	*0.001*^a^	0.378 power: 99.9%
	(+)	9.6 ± 8.8	8 (0‒38)		
**Smoking**	(-)	7.3 ± 8.9	3 (0‒41)	*0.49*	0.018 power: 6%
	(+)	7.4 ± 8.8	5 (0‒44)		
**F.CAD**	(-)	7.3 ± 8.9	4 (0‒44)	*0.72*	0.045 power:5%
	(+)	7.32±8.6	5 (0‒31)		

Mann Whitney *U*-Test.

H.CAD, History of Coronary Artery Disease; F.CAD, Family history of Coronary Artery Disease; DM, Diabetes Mellitus; HT, Hypertension; (-), Absent; (+), Present.

a p < 0.01.

### Sex-specific associations between CVRFs and the localization of CAG lesions

Sex-specific associations between CVRFs and the localization of CAG lesions are presented in (Table 1S) and (Table 2S) for men and in (Table 3S) and (Table 4S) for women, as demonstrated in the Supplement part.

### HT

In patients with HT, severe CAD (> 70%) was observed more frequently in the Cx (p = 0.029; p < 0.05) and the RCA (p = 0.002; p < 0.01) among women (Table 3S). In contrast, in men, severe disease was more commonly observed in the proximal LAD (p = 0.024; p < 0.05) and the mid LAD (p = 0.045; p < 0.05) segment (Table 1S).

Regression analysis performed to identify the risk factors associated with proximal and mid LAD segments was found to be statistically significant for both the proximal LAD segment (*F* = 4794; p = 0.009; p < 0.01) and the mid LAD segment (*F* = 4025; p = 0.008; p < 0.01) in men. The presence of HT increased the risk of proximal LAD stenosis by 0.999 units (β = 0.999; p = 0.039; p < 0.05) and the risk of LAD mid stenosis by 0.115 units (β = 0.115; p = 0.017; p < 0.05) in men (Table 4).

**Table 4:** Independent predictors of stenosis severity in men.

**Dependent Variable**	**Independent Variable**	**β**	***t***	**p**	***F***	**Model (p)**	**R**^2^
LMCA	*Constant*		3.318	***0.001***^a^	7.958	**0.005**^a^	0.018
	**Creatinine**	0.135	2.821	***0.005***^a^			
LAD Prox	Sabit		3.841	***0.001***^a^	4.794	**0.009**^a^	0.022
	**HT**	0.099	2.072	***0.039***^b^			
	**Glucose**	0.106	2.214	***0.027***^b^			
LAD Mid	*Constant*		7.271	***0.001***^a^	4.025	**0.008**^a^	0.027
	**HT**	0.115	2.401	***0.017***^a^			
	H.CAD	0.080	1.664	*0.1*			
	Creatinine	0.089	1.861	*0.06*			
Dia	*Constant*		3.536	***0.001***^a^	3.679	**0.026**^b^	0.017
	**TG**	0.084	1.752	*0.080*			
CX	*Constant*		4.340	***0.001***^a^	9.042	**0.003**^a^	0021
	**Glucose**	0.144	3.007	***0.003***^b^			
RCA	*Constant*		2.731	*0.007*	5.280	**0.001**^a^	0.036
	**H. CAD**	0.095	1.993	***0.047***^b^			
	**Glucose**	0.112	2.318	***0.021***^b^			
	TG	0.093	1.921	*0.055*			

HT, Hypertension; H.CAD, History of Coronary Artery Disease; TG, Triglyceride; LDL-C, Low-density lipoprotein. Method: Backward Linear Regression Analysis.

a p < 0.01.

b p < 0.05.

### DM

A significant association was observed between the presence of DM and the severity of CAD in all coronary vessels except the LMCA in women (LAD prox: p = 0.001; p < 0.01; LAD mid: p = 0.001; p < 0.01; Dg: p = 0.003; p < 0.01; Cx: p = 0.001; p < 0.01; RCA: p = 0.001; p < 0.01; LMCA: p > 0.05) (Table 3S). In men, no significant association was observed between DM and moderate or severe coronary obstruction. However, mild obstruction in CX was more common in men (p = 0.033; p < 0.05) (Table 1S).

The regression analysis performed to identify the risk factors associated with LAD prox, mid LAD, Dg and Cx involvement was found to be statistically significant respectively as (*F* = 15,222; p = 0.001; p < 0.01), (*F* = 7034; p = 0.001; p < 0.01), (*F* = 7260; p = 0.001; p < 0.01), (*F* = 10,381; p = 0.001; p < 0.01) in women. DM increased the risk of proximal LAD stenosis by 0.185 units (β = 0.185; p = 0.001; p < 0.01), mid LAD stenosis by 0.136 units (β = 0.136; p = 0.009; p < 0.01), Dg stenosis by 0.123 units (β = 0.123; p = 0.019; p < 0.05) and Cx stenosis by 0.116 units (β = 0.116; p = 0.025; p < 0.05) in women demonstrating diffuse involvement (Table 5).

**Table 5:** Independent predictors of stenosis severity in women.

**Dependent Variable**	**Independent Variable**	**β**	***t***	**p**	***F***	**Model (p)**	**R**^2^
LMCA	*Constant*		1.677	*0.094*	1.982	**0.160**	0.005
	F.CAD	0.067	1.408	*0.16*			
LAD Prox	*Constant*		3.958	***0.001***^a^	15.222	**0.001**^a^	0.034
	**DM**	0.185	3.901	***0.001***^a^			
LAD Mid	*Constant*		0.802	*0.423*	7.034	**0.001**^a^	0.076
	**DM**	0.136	2.629	***0.009***^a^			
	**Glucose**	0.131	2.537	***0.012***^b^			
	Creatinine	0.089	1.870	*0.06*			
	LDL-C	0.215	1.882	*0.061*			
Dia	*Constant*		1.069	*0.286*	7.260	**0.001**^a^	0.033
	**DM**	0.123	2.350	***0.019***^b^			
	Glucose	0.091	1.731	*0.08*			
CX	*Constant*		−3.080	***0.002***^a^	10.381	**0.001**^a^	0.108
	**DM**	0.116	2.247	***0.025***^b^			
	H.CAD	0.080	1.716	*0.09*			
	**F.CAD**	0.104	2.256	***0.025***^b^			
	**Glucose**	0.123	2.422	***0.016***^b^			
	**Creatinine**	0.202	4.345	***0.001***^a^			
RCA	*Constant*		−0.645	*0.52*	8.822	**0.001**^a^	0.094
	HT	0.084	1.767	*0.08*			
	**H.CAD**	0.100	2.149	***0.032***^b^			
	**Glucose**	0.148	3.151	***0.002***^a^			
	**Creatinine**	0.156	3.300	***0.001***^a^			
	HDL-C	−0.082	−1.757	*0.08*			

HT, Hypertension; DM, Diabetes Mellitus; H.CAD, History of Coronary Artery Disease; F.CAD, Family history of Coronary Artery Disease; TG, Triglyceride; LDL-C, Low Density Lipoprotein; HDL-C, High Density Lipoprotein; Total-C, Total Cholesterol.

Method: Backward Linear Regression Analysis.

a p < 0,01.

b p < 0,05.

### H.CAD

Moderate to severe lesions were more frequently observed in the proximal (p = 0.013; p < 0.05) and mid segments of the LAD (p = 0.021; p < 0.05) as well as in the CX (p = 0.006; p < 0.01) and the RCA (p = 0.016; p < 0.05) in men (Table 1S). In women, moderate to severe lesions were more common in the proximal LAD (p = 0.047; p < 0.05), CX (p = 0.014; p < 0.05) and RCA (p = 0.009; p < 0.01) (Table 3S).

Regression analysis performed to identify risk factors affecting RCA involvement in men and women demonstrated statistically significant associations in both sexes (men: *F* = 5280; p = 0.001; p < 0.01; women: *F* = 8822; p = 0.001; p < 0.01). H.CAD increased the severity of RCA stenosis by 0.115 units (β = 0.095; p = 0.047; p < 0.05) in men and 0.100 units (β = 0.100; p = 0.032; p < 0.05) in women (Tables 4‒5).

### Smoking

No statistically significant association was found between smoking and the localization or severity of CAD in either men (p > 0.05) (Table 1S) or women (p > 0.05) (Table 3S).

### F. CAD

No significant association was observed between F. CAD and the localization or severity of CAD in either men (p > 0.05) (Table 1S) or women (p > 0.05) (Table 3S). However, regression analysis performed to identify risk factors affecting Cx involvement in women revealed a statistically significant association (*F* = 10,381; p = 0.001; p < 0.01). F. CAD was associated with an increase in Cx stenosis by 0.104 units (β = 0.104; p = 0.025; p < 0.05) in women (Table 5).

### Dyslipidemia

#### TG

No statistically significant association was found between TG levels and the localization or severity of CAD in either men or women (p > 0.05) (Table 2S, Table 4S).

#### TC

No significant association was observed between TC levels and the localization or severity of CAD in men (p > 0.05) (Table 2S). In women, however, a weak correlation was observed between TC levels and stenosis limited to the CX (*r* = −0.120; p = 0.012; p < 0.05) (Table 4S). However, this association was not confirmed in linear regression analysis (Table 5).

#### HDL-C

No statistically significant association was found between HDL-C levels and the localization or severity of CAD in men (p > 0.05) (Table 2S).

In women, weak negative correlations were observed between HDL-C levels and stenosis in the mid LAD (*r* = −0.140; p = 0.003; p < 0.01); Dg (*r* = −0.100; p = 0.034; p < 0.01), CX (*r* = −0.154; p = 0.001; p < 0.01) and RCA (*r* = −0.151; p = 0.001; p < 0.01) (Table 4S).

However, further evaluation using regression analysis did not confirm these associations in women (Table 5).

#### LDL-C

No statistically significant association was found between LDL-C levels and the localization or severity of CAD in men (p > 0.05) except for a weak negative correlation with stenosis in the Dg branch (*r* = −0.123; p = 0.009; p < 0.01) (Table 2S). In women, no significant correlation was observed between LDL-C levels and stenosis in any vessel except for the Cx, where a weak negative correlation was detected (*r* = −0.108; p = 0.023; p < 0.05) (Table 4S).

#### Glucose

Each unit increase in blood glucose level was associated with an increased risk of Cx stenosis by 0.123 unit s (β = 0.123; p = 0.016; p < 0.05), mid LAD stenosis by 0.131 units (β = 0.131; p = 0.012; p < 0.05) and RCA stenosis by 0.148 units (β = 0.148; p = 0.002; p < 0.01) in women. In men, each unit increase in blood glucose level was associated with an increased risk of prox LAD stenosis by 0.106 units (β = 0.106; p = 0.027; p < 0.05), CX stenosis by 0.144 units (β = 0.144; p = 0.003; p < 0.01) and RCA stenosis by 0.112 units (β = 0.112; p = 0.021; p < 0.05) (Table 4, Table 5).

### Creatinine

Each unit increase in serum creatinine level was associated with an increased risk of CX stenosis by 0.202 units (β = 0.202; p = 0.001; p < 0.01) and RCA stenosis by 0.156 units (β = 0.156; p = 0.001; p < 0.01) in women.

In men, each unit increase in serum creatinine level was associated with an increased risk of LMCA stenosis by 0.135 units (β = 0.135; p = 0.005; p < 0.01) (Table 4, Table 5).

## Discussion

This study compares sex-based differences in major conventional CVRFs, provides a sex stratified anatomical analysis of conventional CVRFs and their differential impact on both the severity and territorial distribution of epicardial CAD.

Women in our cohort were older and had a higher prevalence of HT and DM in contrast to some previous reports.^16^ This discrepancy may partly reflect country-specific demographic, socioeconomic and lifestyle characteristics.

Consistent with prior studies,^5^^,^^10^ men had a higher prevalence of smoking^17^ and a greater H.CAD. F.CAD was similar between sexes.

Lipid profiles demonstrated higher Total-C, LDL-C and HDL-C levels in women, whereas no significant sex based difference in TG levels was observed, findings consistent with the existing literature.^16^, ^17^, ^18^ Although creatinine levels differed statistically between sexes, the effect size was small and the post hoc power was low.

Women presenting with chest pain are generally reported to have less severe obstructive CAD than men.^19^ Our findings align with this observation: the incidence of intermediate and critical stenosis across all CAs was higher in men.

Importantly, SS were significantly higher in men, indicating a more complex and extensive CAD burden and which is consistent with prior reports.^20^^,^^21^

This finding is particularly notable given that HT and DM were more prevalent in women, risk factors known to exert substantial CV effects in women.^22^^,^^23^

The 2006 report of the US Surgeon General suggests that the simultaneous presence of smoking and another CVRF is estimated to quadruple cardiovascular risk.^24^ Whether the higher prevalence of smoking in men contributes to the marked sex difference in SS, or whether hormonal and biological differences play a more dominant role, remains to be clarified. Evaluating sex differences in SS may improve understanding of sex based phenotypic expression of coronary atherosclerosis.^25^

Previous reports have suggested that women tend to have involvement of less critical coronary segments compared with men.^26^ Our findings demonstrated that clinically important CA segments were involved in women, but obstruction in the LMCA, LAD, RCA and CX was found to be lower in women, consistent with the existing literature.^27^

Furthermore, distinct territorial patterns of CVRF associations were observed.

In men, associations between CVRFs and stenosis were predominantly localized to the LAD territory. Severe CAD in the presence of HT was more frequently observed in the proximal and mid LAD segments. H.CAD was also strongly associated with severe lesions in proximal and mid LAD as well as in Cx and RCA, showing diffuse involvement in men. Regression analysis confirmed these findings.

Smoking history and H.CAD were strongly associated with CAD severity in men, whereas dyslipidemia and DM did not demonstrate significant associations with anatomical localization or stenosis severity.

This LAD predominant pattern may reflect shear stress dynamics, plaque biology, or sex related differences in vascular remodeling.

In contrast, in women, most CVRFs were associated with stenosis severity and anatomical localization in the Cx and RCA territories.

A significant correlation was observed between DM and CAD severity in all vessels except LMCA in women showing diffuse involvement. Regression analysis confirmed DM as a more profound CVRF in women. This observation aligns with emerging evidence that metabolic dysregulation interacts with hormonal milieu to influence vascular inflammation, endothelial dysfunction, and possibly microvascular remodeling. HT was associated with severe CAD (> 70%) in the Cx and RCA in women, although its overall effect appeared more pronounced in men.

In patients with H.CAD, severe CAD lesions were more frequently observed in the proximal LAD, CX, and RCA in women. Regression analysis further confirmed these findings.

Although dyslipidemia is well established as a major contributor to CAD severity^28^^,^^29^ no previous study has comprehensively evaluated its association with both anatomical localization and stenosis severity in a sex specific manner.

In our cohort, Total-C was not associated with stenosis severity or anatomical localization in either sex. In men, HDL-C and LDL-C showed no correlation with stenosis severity or distribution. In women, a weak, but statistically significant negative correlation was observed between HDL-C and stenosis in the mid-LAD, Dg branch, CX, and RCA segments. However, these associations were not retained after multivariable adjustment. This attenuation likely reflects confounding by age and menopausal status as Higher HDL-C levels in women often represent a premenopausal estrogen milieu.

TG levels were not associated with anatomical localization or stenosis severity in either sex.

The observed association between serum creatinine and specific coronary territories, including LMCA involvement in men and RCA/Cx involvement in women, may suggest a potential sex related pattern. However, confirmation in larger prospective studies is required. Chronic kidney disease is known to accelerate vascular calcification and inflammatory remodeling, which may partly explain this observation.^30^

Most currently used CAD risk scores classify women as being at lower risk than men. Furthermore, CAD scoring systems, including the SS, are largely sex-neutral. However, our findings suggest that sex modifies the anatomical expression of conventional CVRFs. A uniform risk model may therefore underestimate risk complexity in women and mischaracterize disease phenotype. Future risk scoring systems should incorporate sex-based differences in the impact of conventional CVRFs across distinct atherosclerotic pathways.

Microvascular CAD warrants particular attention in women, as CVRFs may differentially affect microvasculature disease patterns, as previously reported in the literature.^31^^,^^32^ In this context, DM, which may promote inflammation and fibrosis in both epicardial coronary arteries and the coronary microvasculature, warrants further investigation in women. In the era of precision medicine, evaluating sex- based differences in proteomic and molecular profiles of CAD and coronary microvascular dysfunction is essential, as has been addressed by Liu Y et al.^31^

Women have historically been underrepresented in cardiovascular clinical trials, limiting the evidence base for sex specific therapeutic recommendations. Consequently, the development of sex-specific strategies and evidence-based guideline recommendations for the prevention and management of CAD has been constrained to date.^33^ Ensuring proportional enrollment of women in CV clinical trials remains an unmet need.

In our study, we deliberately achieved a balanced sex distribution, which we believe is essential for advancing precision CV medicine and improving the understanding of sex-based differences in coronary atherosclerosis.

## Conclusion

In this study, we demonstrated that the impact of conventional CVRFs on CAD is not uniform across sexes and follows distinct anatomical patterns. In men, CVRF associations were predominantly localized within the LAD territory, and overall disease burden ‒ as reflected by higher SS ‒ was significantly greater. In contrast, in women, DM emerged as a diffuse and powerful determinant of multivessel involvement, particularly affecting the Cx and RCA.

These findings support the concept that atherosclerosis may progress through sex-specific anatomical and biological pathways. Importantly, traditional CVRFs appear to exert differential territorial effects rather than uniform coronary involvement.

Our results underscore the need for sex-specific risk stratification models and suggest that future predictive tools ‒ potentially enhanced by artificial intelligence ‒ should incorporate anatomical pattern recognition alongside conventional risk profiling. Advancing precision cardiology requires balanced representation of women and a deeper understanding of sex-driven pathophysiology in CAD.

## Strengths & limitations

Our study has several notable strengths. First, most previous studies in this field have been conducted in Western populations; therefore, our analysis of a Turkish cohort provides valuable data by accounting for potential ethnic differences in CV disease in this regard, our findings demonstrate that men in the Turkish population exhibited more severe CA stenosis. Another important strength of our study is the relatively higher proportion of women included, particularly in light of the underrepresentation of women in earlier studies.

In most previous investigations, the primary research focus was to assess sex differences in management strategies and clinical outcomes following CAG. Consequently, sex-based differences in CAG findings were often only briefly reported and detailed analyses of the anatomical localization and severity of CA obstruction were lacking. In contrast, our study provides a comprehensive evaluation of both the localization and severity of CA obstruction and systematically examines their associations with major CVRFs with direct comparisons between men and women.

We acknowledge several limitations of our study, which have all the limitations inherent to registries while also reflecting a real-world population of elective patients referred for CAG. Because all patients were referred for invasive evaluation of CAD, referral bias may be present. Therefore, our findings cannot be extrapolated to the general population.

In addition, CA stenosis was assessed visually, which represents a commonly used and practical approach in routine clinical practice. However, the use of quantitative coronary angiography might have provided more precise measurements of lesion severity.

In our study, neither intravascular imaging nor computed tomographic examination was performed, sex-based differences in coronary plaque morphology could not be assessed. Consequently, associations with specific morphological features of culprit plaques could not be evaluated.

In addition, the applicability of our findings to other ethnic groups or to patients presenting with acute coronary syndromes is limited. Our results are derived from a single-center cohort within a single country and further studies incorporating diverse ethnicities and geographic regions are warranted.

Women in our study were older, with a mean age of 61±10.3 years, indicating that a substantial proportion were postmenopausal. Although we attempted to perform subgroup analyses comparing premenopausal and postmenopausal women, the number of premenopausal participants was insufficient due to the single-center design. Therefore, meaningful comparisons between premenopausal and postmenopausal women could not be conducted.

Multicenter studies with larger sample sizes are needed to enable such subgroup analysis.

In addition to the observational design and single-center nature of the study, the large number of statistical comparisons increases the risk of false positive findings (Type 1 error). Although regression modeling was used to validate major associations, exploratory correlations should be interpreted cautiously.

Finally, residual confounding correlated to the coexistence of other traditional, nontraditional, and female-specific CVRF s cannot be entirely excluded.

## Abbreviations

CAD, Coronary Artery Disease; CVRFs, Cardiovascular Risk Factors; CAG, Diagnostic Coronary Angiography; CABG, Coronary Artery Bypass Grafting; PCI, Percutaneous Coronary Intervention; TC, Total Cholesterol; LDL-C, LDL Cholesterol; HDL-C, HDL Cholesterol; TG, Triglyceride; BMI, Body Mass Index; HT, Hypertension; DM, Diabetes Mellitus; CA, Coronary Artery; LMCA, Left Main Coronary Artery; LAD, Left Anterior Descending artery; Dg, 1st Diagonal branch of LAD; CX, Circumflex artery; RCA, Right Coronary Artery; F. CAD, Family history of Coronary Artery Disease; H.CAD, History of Coronary Artery Disease; SS, Syntax Score.

## Ethics approval and consent to participate

Ethical approval was obtained from the Health Sciences University Hamidiye Scientific Research Committee with the number 22/201. All patients have given consent for participation.

## Authors' contributions

Constructing an idea for research and manuscript: N.K. Planning methodology to reach the conclusion: N.K. Data Collection and/or processing: Y.K, S.D, S.Y, M.Ş, A.E, A.T, İ.Y, ALO, MO, MU. Analysis and/or Interpretation: N.K, Y.K. Literature Review: N.K, Y.K, A.T. Writer: N.K, Y.K.

## Funding

This research did not receive any specific grant from funding agencies in the public, commercial, or not-for-profit sectors.

## Declaration of competing interest

The authors declare no conflicts of interest.

## Data Availability

The datasets generated and/or analyzed during the current study are available from the corresponding author upon reasonable request.
