# Supplementary material for: Sex-specific differences in the anatomical localization and severity of coronary artery disease: differential impact of conventional cardiovascular risk factors
Source: Clinics (Sao Paulo). 2026 Sep 15;81:101149. doi: 10.1016/j.clinsp.2026.101149 (PMC13598581; doi:10.1016/j.clinsp.2026.101149)
Supplement: Supplementary file 1 [file mmc1.docx]

CLINICS-D-24-01359_Supplementary Material

**Supplement Table legends**

**S-Table 1:** Correlation between degree and place of coronary artery stenosis and cardiovascular risk factors in men

**S-Table 2:** Correlation between laboratory parameters and the place of coronary artery obstruction in men

**S-Table 3:** Correlation between degree and place of coronary artery stenosis and cardiovascular risk factors in women

**S-Table 4 :** Correlation between laboratory parameters and the place of coronary artery obstruction in women

**Table 1S** Correlation between degree and place of coronary artery stenosis and cardiovascular risk factors in men.

|  | | **HT** | **DM** | **H. CAD** | **Smoking** | **F.CAD** |
| --- | --- | --- | --- | --- | --- | --- |
| **LMCA** | 0 | 222 (95.7) | 157 (95.2) | 173 (95.1) | 116 (95.9) | 19 (95.0) |
|  | 1 | 4 (1.7) | 4 (2.4) | 4 (2.2) | 3 (2.5) | 1 (5.0) |
|  | 2 | 6 (2.6) | 4 (2.4) | 5 (2.7) | 2 (1.7) | 0 (0) |
|  | p-value | *0. 69* | *0.81* | *0.76* | *0.92* | *0.57* |
|  | *Post Hoc Effect size / Power* | *0.088 / 8.7%* | *0.071 / 6.3%* | *0.112 / 9.1%* | *0.055 / 6.3%* | *0.088 / 8.5%* |
| **LAD prox** | 0 | 124 (53.4) | 87 (52.7) | 95 (52.2) | 73 (60.3) | 13 (65.0) |
|  | 1 | 33 (14.2) | 23 (13.9) | 31 (17.0) | 19 (15.7) | 2 (10.0) |
|  | 2 | 75 (32.3) | 55 (33.3) | 56 (30.8) | 29 (24.0) | 5 (25.0) |
|  | p-value | ***^b^0.024*** | *0.069* | ***^b^0.013*** | *0.301* | *0.946* |
|  | *Post Hoc Effect size / Power* | ***0.255 / 46.6%*** | *0.178 / 12.5%* | ***0.303 / 63.4%*** | *0.187 / 11.5%* | *0.032 / 4.2%* |
| **LAD mid** | 0 | 133 (57.3) | 95 (57.6) | 100 (54.9) | 81 (66.9) | 15 (75.0) |
|  | 1 | 25 (10.8) | 21 (12.7) | 25 (13.7) | 11 (9.1) | 3 (15.0) |
|  | 2 | 74 (31.9) | 49 (29.7) | 57 (31.3) | 29 (24.0) | 2 (10.0) |
|  | p-value | ***^b^0.045*** | *0.211* | ***0.021*** | *0.567* | *0.153* |
|  | *Post Hoc Effect size / Power* | ***0.234 / 39.7%*** | *0.114 / 8.8%* | ***0.277 / 54.4%*** | *0.119 / 6.5%* | *0.387 / 21.5%* |
| **Dg** | 0 | 190 (81.9) | 137 (83.0) | 145 (79.7) | 104 (86.0) | 17 (85.0) |
|  | 1 | 15 (6.5) | 9 (5.5) | 16 (8.8) | 6 (5.0) | 0 (0) |
|  | 2 | 27 (11.6) | 19 (11.5) | 21 (11.5) | 11 (9.1) | 3 (15.0) |
|  | p-value | *0.964* | *0.929* | *0.179* | *0.490* | *0.589* |
|  | *Post Hoc Effect size / Power* | *0.002 / 1.3%* | *0.015 / 1.8%* | *0.128 / 9.5%* | *0.134 / 8.7%* | *0.123 / 10.8%* |
| **CX** | 0 | 125 (53.9) | 81 (49.1) | 89 (48.9) | 69 (57.0) | 11 (55.0) |
|  | 1 | 40 (17.2) | 30 (18.2) | 37 (20.3) | 19 (15.7) | 2 (10.0) |
|  | 2 | 67 (28.9) | 54 (32.7) | 56 (30.8) | 33 (27.3) | 7 (35.0) |
|  | p-value | *0,24* | ***^b^0.03*** | ***^a^0.01*** | *0.93* | *0.81* |
|  | *Post Hoc Effect size / Power* | *0.160 / 19.4%* | ***0.254 / 46.4%*** | ***0.451 / 62.8%*** | *0.009 / 10.4%* | *0.019 / 3.4%* |
| **RCA** | 0 | 117 (50.4) | 79 (47.9) | 83 (45.6) | 68 (56.2) | 12 (60.0) |
|  | 1 | 36 (15.5) | 27 (16.4) | 29 (15.9) | 18 (14.9) | 3 (15.0) |
|  | 2 | 79 (34.1) | 59 (35.8) | 70 (38.5) | 35 (28.9) | 5 (25.0) |
|  | p-value | *0.281* | *0.134* | ***^b^0.016*** | *0.673* | *0.814* |
|  | *Post Hoc Effect size / Power* | *0.158 / 14.6%* | *0.110 / 16.3%* | ***0.276 / 54.2%*** | *0.088 / 8.6%* | *0.158% / 18.9%* |

Fisher Freeman Halton Test, ^a^ p < 0.01, ^b^ p < 0.05.

LAD prox, Left Anterior Descendingartery proximal segment; LMCA, Left Main Coronary Artery; RCA, Right Coronary Artery; Cx, Circumflex artery; Dg, 1^st^ Diagonal artery; HT, Hypertension; DM, Diabetes Mellitus; H.CAD, History of Coronary Artery Disease; F.CAD, Family history of Coronary Artery Disease; 0, No stenosis; 1, Moderate stenosis; 2, Severe (> 70%)stenosis.

**Table 2S** Correlation between laboratory parameters and the place of coronary artery obstruction in men.

|  | | **Glucose** | **Creatinine** | **TG** | **HDL-C** | **Total-C** | **LDL-C** |
| --- | --- | --- | --- | --- | --- | --- | --- |
| **LMCA** | *r* | 0.039 | 0.024 | 0.016 | 0.013 | 0.008 | -0.019 |
|  | p | *0.41* | *0.60* | *0.74* | *0.79* | *0.87* | *0.69* |
|  | *Post Hoc Effect size* | *0.197* | *0.155* | *0.126* | *0.114* | *0.089* | *0.138* |
|  | *Power* | *50.7%* | *33.8%* | *24.1%* | *20.4%* | *14.3%* | *27.8%* |
| **LAD prox** | *r* | 0.109 | 0.027 | 0.032 | 0.034 | 0.021 | -0.011 |
|  | p | ***^b^0.02*** | *0.57* | *0.5* | *0.47* | *0.66* | *0.81* |
|  | *Post Hoc Effect size* | ***0.330*** | *0.164* | *0.179* | *0.184* | *0.145* | *0.105* |
|  | *Power* | ***92.4%*** | *37.4%* | *43.2%* | *45.4%* | *30.2%* | *17.9%* |
| **LAD Mid** | *r* | 0.051 | 0.012 | 0.040 | 0.015 | 0.008 | -0.036 |
|  | p | *0.27* | *0.80* | *0.40* | *0.75* | *0.87* | *0.44* |
|  | *Post Hoc Effect size* | *0.225* | *0.109* | *0.200* | *0.122* | *0.089* | *0.189* |
|  | *Power* | *62.3%* | *19.2%* | *51.8%* | *22.9%* | *14.3%* | *47.6%* |
| **Dg** | *r* | 0.019 | -0.086 | 0.089 | -0.043 | -0.074 | -0.123 |
|  | p | *0.68* | *0.06* | *0.06* | *0.36* | *0.12* | *0.1* |
|  | *Post Hoc Effect size* | *0.137* | *0.293* | *0.298* | *0.207* | *0.272* | *0.351* |
|  | *Power* | *27.8%* | *84.8%* | *86.1%* | *54.8%* | *78.8%* | *95.2%* |
| **CX** | *r* | 0.158 | 0.054 | 0.080 | -0.042 | 0.020 | -0.023 |
|  | p | ***^a^0.001*** | *0.25* | *0.09* | *0.37* | *0.67* | *0.63* |
|  | *Post Hoc Effect size* | ***0.397*** | *0.232* | *0.283* | *0.204* | *0.141* | *0.152* |
|  | *Power* | ***98.6%*** | *64.9%* | *82%* | *53.8%* | *29%* | *32.7%* |
| **RCA** | *r* | 0.136 | 0.034 | 0.098 | -0.094 | -0.012 | -0.044 |
|  | p | ***^a^0.004*** | *0.46* | *0.06* | *0.06* | *0.79* | *0.35* |
|  | *Post Hoc Effect size* | ***0.369*** | *0.184* | *0.313* | *0.306* | *0.109* | *0.209* |
|  | *Power* | ***96.9%*** | *45.4%* | *89.3%* | *88%* | *19.2%* | *55.8%* |

*r*, Spearman's correlation coefficient, ^a^ p < 0.01, ^b^ p < 0.05.

LAD, Left Anterior Descending artery; LDL, Low Density Lipoprotein; LMCA, Left Main Coronary Artery; RCA, Right Coronary Artery; CX, Circumflex Artery; Dg,1^st^ Diagonal artery; TG, Triglyceride; Total-C, Total Cholesterol; HDL-C, High Density Lipoprotein; LDL-C, Low Density Lipoprotein.

**Table 3S** Correlation between degree and place of coronary artery stenosis and CVRFs in women.

|  | | **HT** | **DM** | **H.CAD** | **Smoking** | **F.CAD** |
| --- | --- | --- | --- | --- | --- | --- |
| **LMCA** | 0 | 318 (98.5) | 194 (98.0) | 106 (97.2) | 84 (96.6) | 32 (97.0) |
|  | 1 | 2 (0.6) | 2 (1.0) | 2 (1.8) | 2 (2.3) | 0 (0) |
|  | 2 | 3 (0.9) | 2 (1.0) | 1 (0.9) | 1 (1.1) | 1 (3.0) |
|  | p-value | *0.81* | *0.38* | *0.13* | *0.07* | *0.35* |
|  | *Post Hoc Effect size/ Power* | *0.087/1.3%* | *0.102/8.6%* | *0.205/9.3%* | *0.478/97.4%* | *0.218/32.1%* |
| **LAD prox** | 0 | 271 (83.9) | 156 (78.8) | 86 (78.9) | 76 (87.4) | 25 (75.8) |
|  | 1 | 15 (4.6) | 14 (7.1) | 7 (6.4) | 4 (4.6) | 4 (12.1) |
|  | 2 | 37 (11.5) | 28 (14.1) | 16 (14.7) | 7 (8.0) | 4 (12.1) |
|  | p-value | *0.19* | ***^*^0.001*** | ***^**^0.047*** | *0.83* | *0.06* |
|  | *Post Hoc Effect size/ Power* | *0.218/34.5%* | ***0.459/96.2%*** | ***0.358/70.5%*** | *0.077/7.7%* | *0.450/28.1%* |
| **LAD mid** | 0 | 244 (75.5) | 135 (68.2) | 76 (69.7) | 70 (80.5) | 26 (78.8) |
|  | 1 | 26 (8.0) | 20 (10.1) | 12 (11) | 3 (3.4) | 2 (6.1) |
|  | 2 | 53 (16.4) | 43 (21.7) | 21 (19.3) | 14 (16.1) | 5 (15.2) |
|  | p-value | *0.17* | ***^a^0.001*** | *0.07* | *0.33* | *1.00* |
|  | *Post Hoc Effect size/ Power* | *0.199/29.1%* | ***0.464/96.6%*** | *0.280/55.5%* | *0.201/24.2%* | *0.008/0.7%* |
| **Dg** | 0 | 276 (85.4) | 155 (78.7) | 89 (82.4) | 76 (87.4) | 25 (75.8) |
|  | 1 | 12 (3.7) | 14 (7.1) | 7 (6.5) | 5 (5.7) | 3 (9.1) |
|  | 2 | 35 (10.8) | 28 (14.2) | 12 (11.1) | 6 (6.9) | 5 (15.2) |
|  | p-value | *0.13* | *^a^0.003* | *0.51* | *0.64* | *0.17* |
|  | *Post Hoc Effect size/ Power* | *0.247/30.5%* | *0.658/99.5%* | *0.278/28.9%* | *0.110/11.2%* | *0.303/63.4%* |
| **CX** | 0 | 236 (73.1) | 133 (67.2) | 73 (67.0) | 68 (78.2) | 22 (66.7) |
|  | 1 | 25 (7.7) | 17 (8.6) | 8 (7.3) | 7 (8.0) | 2 (6.1) |
|  | 2 | 62 (19.2) | 48 (24.2) | 28 (25.7) | 12 (13.8) | 9 (27.3) |
|  | p-value | ***^b^0.029*** | ***^a^0.001*** | ***^b^0.014*** | *0.75* | *0.23* |
|  | *Post Hoc Effect size/ Power* | ***0.292/59.6%*** | ***0.574/99.2%*** | ***0.451/78.4%*** | *0.078/4.2%* | *0.322/69.7%* |
| **RCA** | 0 | 231 (71.5) | 132 (66.7) | 71 (65.1) | 69 (79.3) | 25 (75.8) |
|  | 1 | 22 (6.8) | 17 (8.6) | 9 (8.3) | 2 (2.3) | 1 (3.0) |
|  | 2 | 70 (21.7) | 49 (24.7) | 29 (26.6) | 16 (18.4) | 7 (21.2) |
|  | p-value | ***^a^0.002*** | ***^a^0.001*** | ***^a^0.009*** | *0.27* | *0.76* |
|  | ***Post Hoc Effect size/ Power*** | ***0.640/99.2%*** | ***0.757/99.9%*** | ***0.358/80%*** | *0.305/57.7%* | *0.162/19.8%* |

Fisher Freeman Halton Test, ^a^ p < 0.01; ^b^ p < 0.05.

LAD prox, Left Anterior Descending artery proximal; LMCA, Left main Coronary Artery; RCA, Right Coronary Artery; Dg, 1^st^ Diagonal artery; CX, Circumflex artery; HT, Hypertension; DM, Diabetes Mellitus; H.CAD, History of Coronary Artery Disease; F. CAD, Family history of Coronary Artery Disease; 0, No stenosis; 1, Moderate stenosis; 2, Severe stenosis (> 70%).

**Table 4S** Correlation between laboratory parameters and the place of coronary artery obstruction in women.

|  | | **Glucose** | **Creatinine** | **TG** | **HDL-C** | **Total-C** | **LDL-C** |
| --- | --- | --- | --- | --- | --- | --- | --- |
| **LMCA** | *r* | 0.080 | 0.022 | -0.030 | 0.013 | 0.044 | 0.061 |
|  | p | *0.09* | *0.64* | *0.52* | *0.79* | *0.35* | *0.20* |
|  | *Post Hoc Effect size* | *0.283* | *0.148* | *0.173* | *0.114* | *0.209* | *0.247* |
|  | *Power* | *82.1%* | *31.5%* | *40.9%* | *20.4%* | *55.8%* | *70.4%* |
| **LAD prox** | *r* | 0.090 | -0.018 | 0.057 | -0.096 | 0.014 | 0.040 |
|  | p | *0.055* | *0.704* | *0.230* | *0.055* | *0.763* | *0.394* |
|  | *Post Hoc Effect size* | *0.300* | *0.134* | *0.238* | *0.309* | *0.118* | *0.200* |
|  | *Power* | *86.5%* | *26.6%* | *67.3%* | *88.7%* | *21.6%* | *51.8%* |
| **LAD Mid** | *r* | 0.185 | 0.069 | 0.026 | -0.140 | -0.082 | -0.053 |
|  | p | ***^a^0.001*** | *0.14* | *0.58* | ***^a^0.003*** | *0.083* | *0.26* |
|  | *Post Hoc Effect size* | ***0.430*** | *0.262* | *0.161* | ***0.374*** | *0.286* | *0.230* |
|  | *Power* | ***99.5%*** | *75.8%* | *36.3%* | ***97.3%*** | *83%* | *64.1%* |
| **Dg** | *r* | 0.153 | 0.087 | 0.047 | -0.100 | 0.000 | 0.006 |
|  | p | ***^a^0.001*** | *0.06* | *0.32* | ***^b^0.034*** | *0.99* | *0.91* |
|  | *Post Hoc Effect size* | ***0.391*** | *0.295* | *0.217* | ***0.316*** | *0.003* | *0.077* |
|  | *Power* | ***98.3%*** | *85.3%* | *58.7%* | ***89.9%*** | *0.6%* | *11.9%* |
| **CX** | *r* | 0.177 | 0.137 | 0.038 | -0.154 | -0.120 | -0.108 |
|  | p | ***^a^0.001*** | ***^b^0.003*** | *0.424* | ***^a^0.001*** | ***^a^0.012*** | ***^a^0.023*** |
|  | *Post Hoc Effect size* | ***0.342*** | ***0.370*** | *0.195* | ***0.392*** | ***0.346*** | ***0.328*** |
|  | *Power* | ***94.1%*** | ***97.0%*** | *49.7%* | ***98.4%*** | ***94.7%*** | ***92.2%*** |
| **RCA** | *r* | 0.157 | 0.149 | 0.056 | -0.151 | -0.076 | -0.079 |
|  | p | ***0.001***^b^ | ***0.001***^b^ | ***0.238*** | ***0.001***^b^ | *0.108* | *0.097* |
|  | *Post Hoc Effect size* | ***0.396*** | ***0.386*** | *0.236* | ***0.388*** | *0.275* | *0.281* |
|  | *Power* | ***98.5%*** | ***98%*** | *66.5%* | ***98.2%*** | *80%* | *81.5%* |

*r*, Spearman's correlation coefficient, ^a^ p < 0.01; ^b^ p < 0.05.

LAD, Left Anterior Descending; LMCA, Left Main Coronary Artery; RCA, Right Coronary Artery; DG, 1^st^ Diagonal artery; CX, Circumflex artery; TG, Triglyceride; Total-C, Total Cholesterol; HDL-C, High Density Lipoprotein; LDL-C, Low Density Lipoprotein.
